# Supplementary material for: A Study of the Molecular Regulatory Network of VcTCP18 during Blueberry Bud Dormancy
Source: Plants (Basel). 2023 Jul 9;12(14):2595. doi: 10.3390/plants12142595 (PMC10385817; doi:10.3390/plants12142595)
Supplement: Supplementary file 1 [file plants-12-02595-s001.zip › plants-2440978-supplementary.pdf]

Supplementary Material

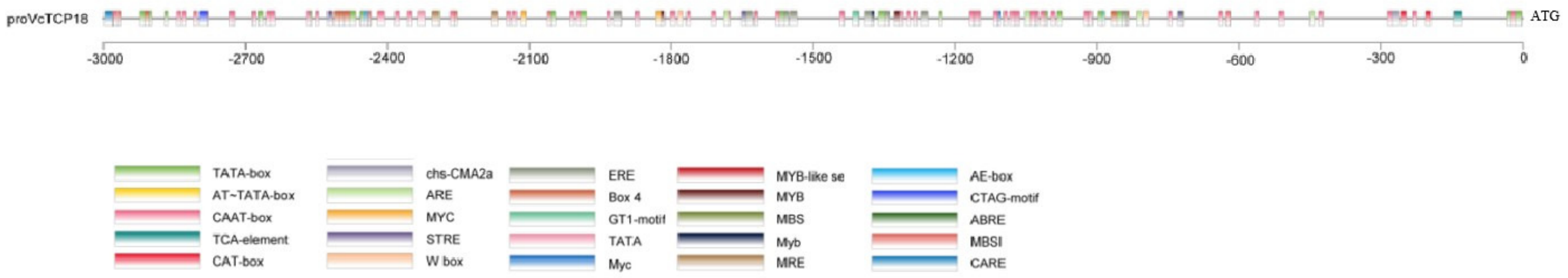

Figure S1.Cis-acting sequence elements of the *VcTCP18* promoter.The different colored rectangles indicated divergent cis-elements.

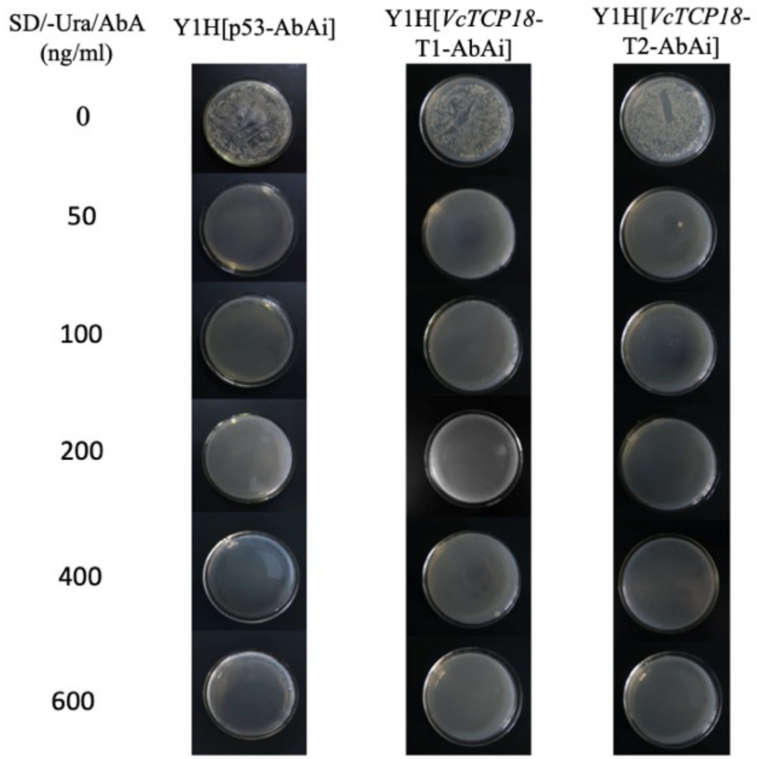

Figure S2. Background level detection.

Table S 1 The primers used in RT-qPCR.

Gene name	Forward primer（5′–3′）	Reverse primer（3′–5′）
VcTCP18	ATCGAGGGATAAAGCAAGAGC	TCACGGCTTCCAGAGTTTG
VcGAPDH	CGGCTACTTACGAGCAAATCAA	TTCAGTGTAGCCCAAAATTCCTTT

Table S2 The primers for cloning *VcTCP18* promoters

Name	Forward primer（5′–3′）	Reverse primer（3′–5′）
pro*VcTCP18-1381Z*	CGGGATCC CACCAAGATTATTGACCGTA	TGCACTGCAG GAGAAAAAGTTGGCTAGAGAG
T1- pAbAi	GGGGTACC GACCGTAAAAAAGGATCTC	GCGTCGAC TGTAAATTGGGTTGGGA
T2- pAbAi	GGGGTACC GGGTTGTTCATTTACTTTCT	GCGTCGAC GGTAGAGGAGTTAGTTCAT
pro*VcTCP18*-LUC 0800	TGCACTGCAG CACCAAGATTATTGACCGTA	CGGGATCC GAGAAAAAGTTGGCTAGAGAG
P1 *VcTCP18*-LUC 0800	TGCACTGCAG AGTTTCTAACAATTGGCAC	CGGGATCC GAGAAAAAGTTGGCTAGAGAG
P2 *VcTCP18*-LUC 0800	TGCACTGCAG GCCTTCTCTCTCTCTCTCTC	CGGGATCC GAGAAAAAGTTGGCTAGAGAG
